# Supplementary material for: Protein Intake Estimated from Brief-Type Self-Administered Diet History Questionnaire and Urinary Urea Nitrogen Level in Adolescents
Source: Nutrients. 2019 Feb 1;11(2):319. doi: 10.3390/nu11020319 (PMC6412261; doi:10.3390/nu11020319)
Supplement: Supplementary file 1 [file nutrients-11-00319-s001.pdf]

Supplementary Materials

Methods

Usual Intake Estimation

Nutrient intake has day-to-day intra-individual variability. True inter-individual variance of usual nutrient intake of a population is smaller than variance calculated from nutrient biomarkers obtained on limited days. When using calculated variance as an index of intake distribution in comparison with dietary references, the proportion of deficiency or excess may be overestimated. We estimated usual intake according to the Nusser's method [1] using PC-SIDE version 1.02 (Iowa State University, Statistical Laboratory, Iowa, USA). Usual intake was compared with the recommended dietary allowance (RDA) [2].

Passing–Bablok Regression

Two measurement variables with measurement errors were compared using the Passing–Bablok regression [3]. This method overcomes weaknesses that ordinal least-square regression has: including assumptions of deterministic *X*, a normal distribution of *Y*, and homogeneity of variance of measurement errors, and instability to outliers. We used *mcreg* in the *mcr* package in *R* [4], where confidence limits were calculated with a bootstrap method, and obtained slopes and intercepts of regression lines with confidence limits between urinary UCr (creatinine) and estimated UCr, between PRT(protein intake)$_{24d}$ and PRT_{24m}, between PRT_{24d} and PRT_{bdhq}, and between PRT_{24m} and PRT_{bdhq}. Estimated UCr was calculated using the Tanaka's equation (see the main text and Appendix A) [5].

Results

Usual Intake Estimation

When the usual protein intake (based on the estimates of protein intake from urinary biomarkers) were calculated, 13.6–19.3% of males, and 22.9–27.8% of females were found to have insufficient protein intake (Table S1).

Table S1. Usual protein intake (g/d) and percentages of individuals below the recommended dietary allowance (RDA).

	Males		Females	
	**Mean g/d ± SD**	**Below RDA (% ± SE)**	**Mean g/d ± SD**	**Below RDA (% ± SE)**
PRT_{24h}	78.6 ± 17.3	13.6 ± 14.7	66.5 ± 15.0	22.9 ± 9.9
PRT_{on}	76.3 ± 18.6	19.3 ± 4.7	64.7 ± 15.4	27.8 ± 4.8

PRT_{24h} and PRT_{on} (protein intake estimated from biomarkers measured in 24-h and overnight urine, respectively), RDA (recommended dietary allowance: 60 g/d for boys, and 55 g/d for girls), SD (standard deviation), SE (standard error of estimate).

Passing–Bablok regression

Slopes of the regression between UCr_{24h} and UCr_{tanaka}, and between PRT_{24h} and PRT_{on} were close to 1, and the confidence intervals did not include zero (Table S2; Figure S1). Slopes of the

regression between PRT_{24h} and PRT_{bdhq}, and between PRT_{on} and PRT_{bdhq} were much larger than 1, and the regression lines were more upward, apart from the identity lines at larger values.

Table S2. Slopes and intercepts of Passing–Bablok regression.

Reference test variables	Slope (lower CL, upper CL)	Intercept (lower CL, upper CL)
UCr_{24h} and UCr_{tanaka}	0.989 (0.669, 1.456)	9.201 (−428.633, 346.385)
PRT_{24h} and PRT_{on}	0.985 (0.709, 1.297)	4.687 (−16.448, 22.512)
PRT_{24h} and PRT_{bdhq}	1.960 (−2.352, 3.147)	−55.838 (−138.617, 243.592)
PRT_{on} and PRT_{bdhq}	2.136 (1.710, 2.722)	−66.811 (−108.074, 38.020)

UCr, urinary creatinine; PRT, protein; 24h, 24-h urine collection; on, overnight urine; CL, confidence limit.

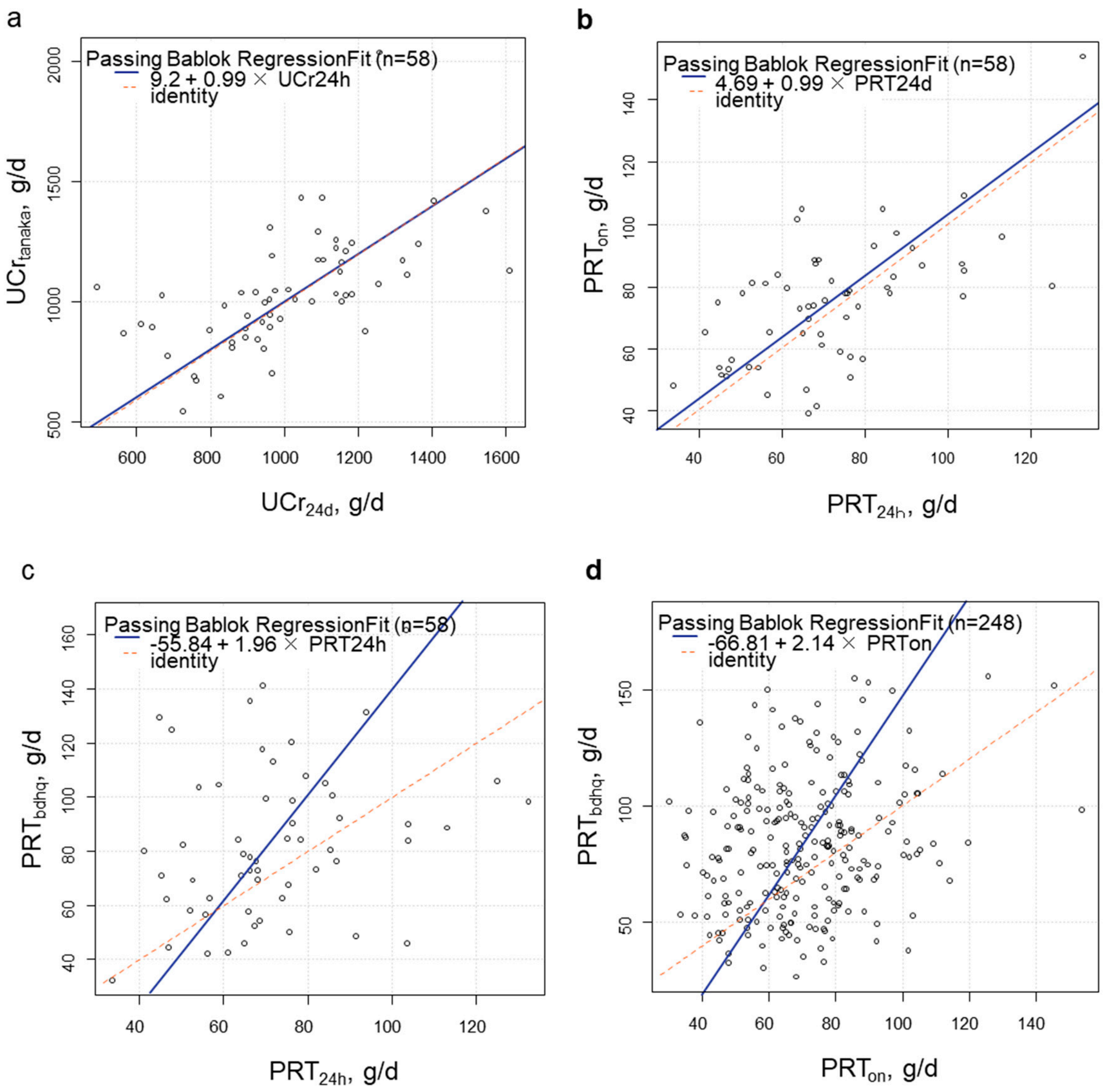

Figure S1. Passing–Bablok regression lines on scatter plots of urinary creatinine or protein intake. A solid line is a regression line with an intercept + a slope, and a dashed line is an identity of the reference. UCr, urinary creatinine; PRT, protein; 24h, 24-h urine collection; on, overnight urine; CL, confidence limit.

References

1. Nusser, S.M.; Carriquiry, A.L.; Fuller, W.A. A semiparametric transformation approach to estimating usual daily intake distributions. *J Am Stat Assoc* **1996**, *91*, 1440-1449, doi: 10.1080/01621459.1996.10476712.
2. Passing, H.; Bablok. A new biometrical procedure for testing the equality of measurements from two different analytical methods. Application of linear regression procedures for method comparison studies in clinical chemistry, Part I. *J Clin Chem Clin Biochem* **1983**, *21*, 709-720.
3. Manuilova E, Schuetzenmeister A. Fabian Model (2014). mcr: Method Comparison Regression. R package version 1.2.1. https://CRAN.R-project.org/package=mcr (accessed on 23 Jan 2019).
4. Hishida, A.; Sasaki, S. Dietary Reference Intakes for Japanese, 2015. Daiichi-Shuppan Co.Ltd.: Tokyo, **2014**, ISBN: 978-4-8041-1312-8 (in Japanese).
5. Tanaka, T.; Okamura, T.; Miura, K.; Kadowaki, T.; Ueshima, H.; Nakagawa, H.; Hashimoto, T. A simple method to estimate populational 24-h urinary sodium and potassium excretion using a casual urine specimen. *J Hum Hypertens* **2002**, *16*, 97-103, doi: 10.1038/sj/jhh/1001307.
